# Cultural Competence and Global Health: Perspectives for Medical Education – Position paper of the GMA Committee on Cultural Competence and Global Health

**DOI:** 10.3205/zma001174

**Published:** 2018-08-15

**Authors:** Claudia Mews, Sylvie Schuster, Christian Vajda, Heide Lindtner-Rudolph, Luise E. Schmidt, Stefan Bösner, Leyla Güzelsoy, Frank Kressing, Houda Hallal, Tim Peters, Margarita Gestmann, Linn Hempel, Tatjana Grützmann, Erika Sievers, Michael Knipper

**Affiliations:** 1 University Medical Center Hamburg-Eppendor,f Department of General Practice/Primary Care , Hamburg, Germany; 2 University Hospital Basel, Head of Program on Diversity Management, Basel, Switzerland; 3 Medical University of Graz, Department of Medical Psychology and Psychotherapy, Graz, Austria; 4 University Medical Center Hamburg-Eppendorf, Center for Psychosocial Medicine, Institute and Outpatients Clinic Medical Psychology, Research Group on Migration and Psychosocial Health (MiPH), Hamburg, Germany; 5 University of Greifswald, Department of Psychiatry and Psychotherapy, Greifswald, Germany; 6 Helios Hanseklinikum Stralsund, Department of Psychiatry and Psychotherapy, Stralsund, Germany; 7 University of Marburg, Department of General Practice/Family Medicine, Marburg, Germany; 8 Paracelsus Medical Private University, Nuremberg Hospital, Department of Psychosomatic Medicine and Psychotherapy, Psychosomatic Consultation and Liaison Service, Nuremberg, Germany; 9 Ulm University, Institute of the History, Philosophy and Ethics of Medicine, Ulm, Germany; 10 University of Cologne, Faculty of Medicine, Cologne, Germany; 11 Ruhr-University Bochum, Medical Faculty, Center for Medical Education, Bochum, Germany; 12 University of Duisburg-Essen, Medical Faculty, Dean's office for student affairs, Essen, Germany; 13 University of Dusseldorf, Medical Faculty, Psychosomatic and Psychotherapy, Dusseldorf, Germany; 14 RWTH Aachen University, Dean's office for student affairs, Aachen, Germany; 15 Academy of Public Health Services, Düsseldorf, Germany; 16 Justus Liebig University Giessen, Institute for the History of Medicine, Giessen, Germany

**Keywords:** cultural competence, global health, migration, medical education, teaching, curriculum

## Abstract

**Introduction: **Routine medical care in Germany, Austria and Switzerland is being increasingly impacted by the cultural and linguistic diversity of an ever more complex world. Both at home and as part of international student exchanges, medical students are confronted with different ways of thinking and acting in relation to health and disease. Despite an increasing number of courses on cultural competence and global health at German-speaking medical schools, systematic approaches are lacking on how to integrate this topic into medical curricula.

**Methodological approach: **This paper is based on a structured consensus-building process by a multidisciplinary committee composed of faculty and students. In a first step, a qualitative online survey was carried out in order to establish an inventory of definitions and concepts. After the second step, in which a literature search was conducted and definitions of global health and transcultural and intercultural competence were clarified, recommendations were formulated regarding content, teaching and institutional infrastructure. Based on small-group work and large-group discussions, different perspectives and critical issues were compiled using multiple feedback loops that served to ensure quality.

**Results:** An inventory on the national and international level showed that great heterogeneity exists in regard to definitions, teaching strategies, teaching formats and faculty qualification. Definitions and central aspects considered essential to medical education were thus established for the use of the terms “cultural competence” and “global health”. Recommendations are given for implementation, ranging from practical realization to qualification of teaching staff and education research.

**Outlook:** High-quality healthcare as a goal calls for the systematic internationalization of undergraduate medical education. In addition to offering specific courses on cultural competence and global health, synergies would be created through the integration of cultural competence and global health content into the curricula of already existing subject areas. The NKLM (the national competence-based catalogue of learning objectives for undergraduate medical education) would serve as a basis for this.

## Zusammenfassung

**Einführung: **Der medizinische Arbeitsalltag in Deutschland, Österreich und der Schweiz ist zunehmend durch kulturelle und sprachliche Diversität in einer komplexer werdenden Welt geprägt. Sowohl im Rahmen von Auslandsaufenthalten als auch im Inland kommen Medizinstudierende mit unterschiedlichen und ihnen teilweise fremd erscheinenden Denk- und Handlungsgewohnheiten bezogen auf Krankheit und Gesundheit in Berührung. Trotz einer steigenden Anzahl an Lehrangeboten zu den Themen Kulturelle Kompetenz und Global Health an den Fakultäten mangelt es bisher an systematischen Ansätzen zur Integration dieser Themenbereiche in die medizinischen Curricula.

**Methodisches Vorgehen: **Das Papier basiert auf einem strukturierten Konsensfindungsprozess durch ein multidisziplinäres Gremium aus Lehrenden und Studierenden. Mittels einer qualitativen Online-Umfrage wurde in einem ersten Schritt eine Bestandsaufnahme zu Definitionen und Konzepten durchgeführt. Nach einer im zweiten Schritt erfolgten Literaturrecherche und Begriffsklärung zu den Bereichen Global Health und Trans- und Interkulturelle Kompetenz wurden in einem kontinuierlichen Arbeitsprozess inhaltliche, didaktische und strukturelle Empfehlungen erarbeitet. Über Kleingruppenarbeit und Plenumsdiskussionen wurden mit mehreren, der Qualitätssicherung dienenden Feedback-Schleifen unterschiedliche Perspektiven und kritische Standpunkte erarbeitet.

**Ergebnisse:** Eine Bestandsaufnahme im nationalen und internationalen Raum ergab, dass eine große Heterogenität z.B. im Hinblick auf Definitionen, Lehrkonzepte und –formate oder die Qualifikation der Lehrenden besteht. Für die Nutzung der Begriffe Kulturelle Kompetenz und Global Health wurden daher für die medizinische Ausbildung als essentiell erachtete Definitionen und zentrale Aspekte benannt. Als Hilfe für die konkrete Umsetzung in der Lehre wurden Empfehlungen von der praktischen Umsetzung über die Qualifikation der Lehrenden bis hin zur Lehrforschung formuliert.

**Ausblick:** Bei dem Ziel einer qualitativ hochwertigen Patientenversorgung besteht Bedarf an einer systematischen Internationalisierung des humanmedizinischen Studiums. Neben der Bereitstellung von spezifischen Lehrangeboten geht es im Sinne der Synergiebildung um eine Integration der Förderung Kultureller Kompetenz und der Vermittlung von Global Health-Inhalten in Angebote bestehender Fachdisziplinen. Der Nationale Kompetenzbasierte Lernzielkatalog Medizin (NKLM) bietet dafür eine Grundlage.

## 1. Introduction

Routine medical care in hospitals, medical practices and other healthcare institutions has increasingly been affected by cultural and linguistic diversity for years.

The profound significance of cultural aspects, as well as of the social, legal and political framework for patient health and medical practice have become increasingly conspicuous in Germany, Austria and Switzerland following the development of globalization and migration processes. Current challenges related to the reception of large numbers of refugees from war zones and regions of conflict [1] dramatically testify to this. Medical students are not only confronted with different and sometimes foreign ways of thinking and acting as relates to disease and health in the context of international student exchanges. Students also experience many different and sometimes unexpected forms of doctor-patient relationships in their native countries. 

It must be taken into consideration, for instance, that wide diversity in as much regards as the understanding of hierarchies, communication styles, the inclusion of family members, or the value of religion and spirituality is not limited solely to contact with patients with migration background. In consequence, cultural competence and global health are indeed relevant to medical practice and decision-making both at home and abroad.

Embedding these topics in medical education is a basic prerequisite in order to ensure high-quality, individualized healthcare for all patients in times of globalization. Furthermore, it is essential to prevent misunderstandings and avoid inappropriate care. Systematically structured and sustainable institutionalized approaches are currently lacking.

Already in 2009, the *Bundesvertretung der Medizinstudierenden in Deutschland* bvmd (the national association of medical students in Germany) pointed out the need to pay stronger attention to global health in medical education [2]. In 2014, the Lancet Commission on Culture and Health emphasized the importance of a broad and nuanced understanding of culture in medicine and fostering the development of cultural competence during medical education [3]. In 2015, the Leopoldina and other scientific academies took a stand on strengthening public health and global health in Germany. They therein articulated a clear recommendation for the exhaustive inclusion of this issue in medical education [4]. Most recently, in 2017, the *Hochschulrektorenkonferenz* (the association of German universities and higher education institutions) issued recommendations for the internationalization of curricula, calling for the systematic integration of courses on cultural competence and global health into medical curricula [5], [6].

Through this position paper, the Committee on Cultural Competence and Global Health of the *Gesellschaft für Medizinische Ausbildung *(GMA) intends to contribute to the systematic development of courses and programs dealing with the tightly interconnected topics of cultural competence and global health in medical education. In addition to an overview of the current situation in German-speaking countries and internationally, the committee presents definitions, theoretical considerations and recommendations regarding teaching and faculty qualification.

## 2. Methodological approach

This paper is the product of a structured consensus-building process [7] which was undertaken from 2013 to 2016. A multidisciplinary committee strategically composed of highly experienced faculty participated in this process. The members represented the diverse academic and professional contexts of clinical medicine, public health, cultural and social sciences and other disciplines relevant to the topic in question. Undergraduate medical students were actively involved alongside faculty. 

The aim was to compile well-balanced and meaningful results and reach a consensus whilst drawing on the multidisciplinary nature of the committee and following a structured process (see Attachment 1).

As initial step, a qualitative online survey was carried out in order to assess pre-existing understandings, definitions and concepts related to the subject area among the 16 original committee members with expertise in teaching intercultural and transcultural competence and/or global health. Based on the results, priorities were set for the next steps in the process. Divided into two working groups, definitions for the terms “global health” and “transcultural/intercultural competence” were elicited based on targeted literature search and extensive deliberations. Recommendations were compiled in an ongoing process concerning content, didactics and structures. The use of different settings such as small-group work, large-group discussions and teleconferences served to identify and discuss different perspectives and critical standpoints while providing feedback loops for quality assurance. A major goal was to develop integrated perspectives regarding topics that are usually viewed separately, such as, for example, transcultural and intercultural competence or cultural competence and global health in medical education.

## 3. Situation in German-speaking countries and internationally

In German-speaking countries, there exists a wide variety of initiatives and programs fostering cultural competence and teaching global health in medical education [2], [8], [9], [10], [11], [12], [13], [14], [15], [16]. At some universities, individual aspects are addressed (e.g. cultural competence or topics regarding the global health spectrum), at others, integrated formats are offered. At many universities, courses are optional, offered voluntarily by committed teaching faculty and particularly motivated students. Structural integration into official curricula is rare [11], [17]. A recent publication on Germany’s increasing role in global health criticized German medical schools for placing low priority on this particular topic [18], [19].

With the introduction in 2015 of the NKLM (the national competence-based catalogue of learning objectives for undergraduate medical education) learning objectives covering the central aspects of cultural competence and global health now exist for Germany [http://www.nklm.de]. The 2008 Swiss Catalogue of Learning Objectives has been revised and replaced by the Profiles Framework (PROFILES 2017) which also contains learning objectives on this topic [http://www.profilesmed.ch/doc/Profiles_2017.pdf]. In Austria, there is no national catalogue of learning objectives yet. A profile required for faculty teaching cultural competence and/or global health does not exist in any of the three countries, because there are no binding requirements regarding content and/or structures for course planning.

By contrast, there has been a decades-long tradition of offering courses and employing elaborate concepts to impart cultural competence during medical education in conjunction with far-reaching academic debates and research in education at some Anglo-American universities [20], [21], [22], [23], [24], [25], [26], [27], [28]. Cultural competence in healthcare has become more important in Europe in recent years as a response to different initiatives, as are, for instance, the EU project on Migrant-Friendly Hospitals (2002-2004) [29] and the national strategy addressing migration and health on the part of the Federal Swiss Health Agency (2002-2017). This increased importance is reflected in the growing numbers of related courses or discussions about course content, structure, teaching methods and faculty qualification [30], [31], [32], [33], [34], [35]. There are also publications about courses and programs on other continents [36], [37]. This likewise applies to the somewhat more recent topic of global health, which is also receiving growing attention in medical education, accompanied by related discussions regarding content, objectives, structure and teaching strategies [14], [38], [39], [40], [41], [42], [43], [44], [45], [46], [47]. However, a general statement about the type of courses or their mere existence in different countries cannot be made, since no comprehensive data has been collected and there are no universal definitions, nor is there uniformity among course titles.

## 4. Definitions and central aspects

Many different ideas and assumptions are associated with the terms “cultural competence” and “global health”, resulting in a diverse range of possible interpretations and understandings. In the course of the consensus-building process, the following points have been identified as central to medical education:

### Global health

The term “global health” designates a broad and heterogeneous subject area characterized by a general interest in the various challenges and potentials of medicine and healthcare in an increasingly complex world [12], [14], [17], [44], [48], [49]. In conjunction, the following three core elements form a working definition of global health and constitute an innovative and necessary perspective for medical education.

- **Health as a human right: **The normative basis for global health lies in the individual human right to the highest attainable standard of health, as stated in Article 12 of the International Covenant on Economic, Social and Cultural Rights adopted by the United Nations [49], [50], [51]. Global health is explicitly based on the human rights concept of equity [48]: All people are equal in regard to dignity and rights, regardless of their origin and all biological, social or other specific differences. The promotion and guarantee of equal rights requires leveling the field in terms of disadvantages and protection against preventable health risks and all forms of discrimination [50], [52].
- **Global perspective: **Global health perspective focuses on the entire world, meaning that it is not limited to specific areas or regions [48], [53]. This involves consideration of local situations within a global context, including migration, climate change, and global economic relationships [15], [54]. The isolated view of medicine and healthcare in terms of the North-South divide, implying supposedly developed and underdeveloped countries, is inappropriate from a global health perspective.
- **Interdisciplinarity: **Global health is an interdisciplinary field [48], [49], [55]. Alongside scientific and clinical concerns, there are also epidemiological, socio-cultural, economic, ecological, ethnic, political and legal contexts relevant to healthcare and the practice of medicine.

#### Cultural competence

Against the backdrop of socio-cultural diversity of populations, culture is an ambivalent and complex term, even in the context of medicine and healthcare [10], [21], [22], [23], [25], [56], [57]: Careless use of the term can lead to stereotyping and misunderstandings, while deeper reflection on the term’s meaning offers the chance for better understanding and improved interaction with all patients. A more differentiated and culturally aware view places the focus on patients’ reality and the concrete significance of social, cultural and structural aspects of health, medicine and healthcare [20], [21], [58], [59], [60].

This complexity has led to a wide variety of theoretical views, concepts and terms in the academic debate. For instance, there are numerous definitions of the terms “interculturality” and “transculturality” [8], [25], [59], [61], [62], [63], [64]. The aims and main aspects of intercultural and transcultural competence derived from these terms are not contradictory, but rather exhibit large overlap and can mutually supplement each other. Based on the consensus reached, the term “cultural competence” is recommended, as it is to be understood in a comprehensive, overarching manner and is defined for use in medical education by the following aspects:

- An understanding of culture based on socio- and cultural anthropology, which views culture as a collection of ways of thinking and acting acquired in a lifetime and acknowledges cultural identity depends on context [21].
- A nuanced and self-critical reflection of the physician’s own socialization, individual culture and stereotypes.
- Awareness of patients as individuals, and reflection on both the aspects perceived to be culturally different and shared commonalities.
- The ability to assess the linguistic proficiency of individual patients along with knowing the advantages and disadvantages of using professional or non-professional interpreters to communicate with patients.
- Attentiveness to the patients’ prior experiences regarding healthcare in their country of origin and abroad, to possible migration backgrounds and experiences therein, as well as to individual concepts regarding health, disease and therapy.
- Critical reflection on beliefs, patterns of thought and action, as well as the hierarchies of values held by the medical personnel in medical schools, hospitals and medical practices [3], [64].
- Systematic consideration of the social, economic, political and structural factors influencing medical care, in particular as regards alleged “cultural” challenges [23], [28].

These central aspects contradict the frequently expressed desire for simple checklists and rules of conduct for certain cultures and medical situations. Culturally competent care for patients can only be ensured through constant communication and comprehension of the priorities and needs of those involved.

## 5. Recommendations

The following recommendations are made based on the above definitions and theoretical considerations, the teaching experience of the experts involved in reaching the consensus-building process, and relevant international publications.

1. **Utilization of synergies by linking cultural competence with global health in medical education: **The advantages of linking these two areas, though they are often considered and taught separately, arise from the important overlap of topics and ideas. Topics relevant to both include migration, diversity or the necessity of a broadly conceived interdisciplinary perspective. In addition, students’ international experiences connected to a global health perspective (e.g. specialized studies abroad) offer a wide variety of didactically useful interaction as regards cultural competence. While teaching skills related to cultural competence is crucial to courses on global health, aspects of global health are not absolutely necessary in courses dealing with cultural competence, though they are definitely desirable.
2. **Embedding topics of cultural competence and global health in the curricula: **Due to their relevance to the routine of medical care, cultural competence and global health should be integrated into the standard curricula and required from all medical students. Longitudinal embedding in the curriculum with consideration of university-specific pre-requisites and structures should be aimed for. Sufficient staffing and funding is to be ensured. In-depth courses for particularly motivated students are recommended.
3. **Faculty qualification and teaching methodology: **In addition to the above definitions and core content, individual commitment to the objectives, content and theoretical background of these concepts is an important pre-requisite for teaching cultural competence and global health. The ability to adequately analyze the root causes and consequences of diversity is essential, as is systematic reflection on one’s own attitude and actions. Professionalization of faculty and the development of criteria for quality assurance are also required. In terms of didactics, conventional approaches should be complemented by methods involving the students’ individual experiences and backgrounds, e.g. intercultural experiences in their personal lives, daily clinical practice, or participation in international student exchange projects.
4. **Interdisciplinary approach:** Cultural competence and global health embody a broad interdisciplinarity that combines the study of culture, society, law, politics, religion, economics, medical ethics and other disciplines. Justice will need to be done to this cross-disciplinary aspect when designing a curriculum, since the inclusion of the social, economic, political and cultural determinants in global healthcare is a major goal of teaching cultural competence and global health. Cultural competence and global health thus combine to form a subject area that reflects in a very special manner the overall mandate of higher education to foster comprehensive academic learning.
5. **Research in education: **Concurrent research in education and evaluation are a necessary accompaniment to the continued development and optimization of this interdisciplinary topic currently undergoing development and taking form in a plethora of different courses and teaching approaches.

## 6. Outlook

High-quality healthcare as a goal calls for the systematic internationalization of undergraduate medical education. In addition to offering specific courses on cultural competence and global health, synergies would be created through the integration of cultural competence and global health content into the curricula of already existing subject areas. 

The NKLM [http://www.nklm.de] adopted in 2015 represents an important basis for integrating cultural competence and global health into existing curricula. As this is accomplished, it will be important to consider university-specific structures and individual national characteristics and to strengthen institution-level structures.

Furthermore, the internationality connected with cultural competence and global health aligns with the lives and future plans of many students. Even without intending to practice medicine abroad, many students seek opportunities to foster and develop their social, cultural and often humanitarian interests and skills during their studies and later as practicing medical professionals. This aim is served by anchoring this topic more strongly into undergraduate medical education.

The committee will be happy to answer any questions or take suggestions.

## Notes

This is the English translation of the article originally written in German. 

The references have been compiled by the committee as a list of publications relevant to this position paper. This selection intends by no means to be an exhaustive one.

The position paper was accepted by the GMA executive board on June 11, 2018.

## Competing interests

The authors declare that they have no competing interests. 

## Supplementary Material

Questions for (decentralized) preparation of the Retreat (virtual platform, March 2014)
