# Supplementary material for: Cultural Competence and Global Health: Perspectives for Medical Education – Position paper of the GMA Committee on Cultural Competence and Global Health
Source: GMS J Med Educ. 2018 Aug 15;35(3):Doc28. doi: 10.3205/zma001174 (PMC6120152; doi:10.3205/zma001174)
Supplement: Questions for (decentralized) preparation of the Retreat (virtual platform, March 2014) [file JME-35-28-s-001.pdf]

GMA Committee on Intercultural Competence & Global Health

Questions for (decentralized) preparation of the Retreat (virtual platform, March 2014)

Aims

- Inclusion of all committee members in the development of concepts and content (regardless retreat attendance)
- Transparency and efficiency of committee work
- The broadest and most comprehensive collection possible of suggestions and ideas, theoretical and conceptual approaches, issues and critical comments on the topics of cultural competence and global health in medical education.
- Provide a basis for the retreat on March 28 – 30, 2014 in Gießen

Methodology

- Open questions with sufficient space for precise answers, explanations, including recommended literature
- Anonymous collection and analysis, in which all responses to the questions will be compiled and made available to the active members (as PDF file).
- Use of an online survey tool (no GMA Internet platform available to date): https://de.surveymonkey.com/s/8KB26KQ
- Time period for the survey: March 12 – 23, 2014. On March 25 the unfiltered results will be sent as a PDF to all active members (“active”: active response to committee work or retreat [commit to attend or not attend] since committee founding in 2013).

Technical aspects

- Responses will be registered via the computer that was used (IP address will be saved for this purpose), so that the survey can be ended at any time and continued later on the same computer (one survey per computer).
- Data transmission is not SSL-secure to avoid problems with the university/institution firewalls. This is fine given the nature of the data being shared.

Questions

The questions are divided into seven blocks and numbered sequentially. It is not expected that all questions will receive in-depth and complete responses. It is all voluntary! Here are all the questions:

1) Definition of terms: Intercultural and transcultural competence
   1. Which definitions/aspects of the term “intercultural competence” are familiar to you? (if possible, please cite sources)
   2. In your opinion what does the term “intercultural competence” encompass? (free association, including keywords)
   3. Which definitions/aspects of the term “transcultural competence” are familiar to you? (if possible, please cite sources)
   4. In your opinion what does the term “transcultural competence” encompass? (free association, including keywords)
   5. Which term do you prefer? Which advantages and disadvantages do you see in the designations?

6. Which definitions do you use in your own work?

2) Definitions of the term: Global Health
   7. Which definitions/aspects of the term “global health” are familiar to you? (if possible, please cite sources)
   8. In your opinion what does the term “global health” encompass? ((free association, including keywords)
   9. Which definitions do you use in your own work?

3) Connection between intercultural competence & global health
   When you think of intercultural competence and global health as topics in medical education:
   10. Where do you see overlap and connections between the two topics?
   11. What separates the two topics?
   12. What is specific to intercultural competence?
   13. What is specific to global health?
   14. Which arguments support addressing these two topics together in medical education?
   15. Which arguments speak against doing this? (For instance, do you see topics or aspects that go missing or could be neglected in the very comprehensive topic of intercultural competence and global health?)

4) Two questions regarding the GMA committee
   16. On what should our committee place its priority?
   17. What should the committee accomplish? What are the goals?

5) Literature proposals
   18. Please indicate (5 – 6 max.) scientific articles on theoretical, conceptual or pedagogical topics which you find particularly significant (including partial aspects of intercultural competence and global health); ideally with an explanation for your recommendation.

6) Personal experience / Teaching experience
   19. Do you teach one of these topics? If yes, which one(s)?

7) Final questions
   20. What do you consider to be the most important challenges for teaching medicine related to this context?
   21. Do you have any additional comments, suggestions or questions for the committee or those attending the retreat (March 26-28)?
